# Generation of Self-Assembled 3D Network in TPU by Insertion of Al_2_O_3_/*h*-BN Hybrid for Thermal Conductivity Enhancement

**DOI:** 10.3390/ma14020238

**Published:** 2021-01-06

**Authors:** Kai-Han Su, Cherng-Yuh Su, Po-Wei Chi, Prem Chandan, Cheng-Ta Cho, Wan-Yu Chi, Maw-Kuen Wu

**Affiliations:** 1 Institute of Mechatronic Engineering, National Taipei University of Technology, 1, Section 3, Zhongxiao E. Road, Taipei 106, Taiwan; kelvian303@gmail.com (K.-H.S.); jocelyn841026@gmail.com (W.-Y.C.); 2 Institute of Physics, Academia Sinica, 128, Section 2, Academia Road, Taipei 115, Taiwan; jacky01234567891@hotmail.com (P.-W.C.); coorgchandu@gmail.com (P.C.); mkwu@phys.sinica.edu.tw (M.-K.W.); 3 Additive Manufacturing Center for Mass Customization Production, National Taipei University of Technology, 1, Section 3, Zhongxiao E. Road, Taipei 106, Taiwan; jimmycho10245@mail.ntut.edu.tw

**Keywords:** TPU, Al_2_O_3_/*h*-BN hybrid, thermal interface materials (TIMs), self-assembled 3D network, thermal conductivity

## Abstract

Thermal management has become one of the crucial factors in designing electronic equipment and therefore creating composites with high thermal conductivity is necessary. In this work, a new insight on hybrid filler strategy is proposed to enhance the thermal conductivity in Thermoplastic polyurethanes (TPU). Firstly, spherical aluminium oxide/hexagonal boron nitride (ABN) functional hybrid fillers are synthesized by the spray drying process. Then, ABN/TPU thermally conductive composite material is produced by melt mixing and hot pressing. Then, ABN/TPU thermally conductive composite material is produced by melt mixing and hot pressing. Our results demonstrate that the incorporation of spherical hybrid ABN filler assists in the formation of a three-dimensional continuous heat conduction structure that enhances the thermal conductivity of the neat thermoplastic TPU matrix. Hence, we present a valuable method for preparing the thermal interface materials (TIMs) with high thermal conductivity, and this method can also be applied to large-scale manufacturing.

## 1. Introduction

In this modern technological era, electronic gadgets and devices play a major role in every field. The electronics industry has been incessantly focusing on designing high performing, efficient, miniaturized, and cost-effective electronic products. Apart from the above criteria, thermal management also plays a critical part in designing electronic products. The excess irrelevant heat produced by the devices can get accumulated, affecting the operational efficiency and reliability of the devices [1]. Therefore, thermal interface materials (TIM) play a vital role in enhancing heat transfer between heat sources and heat sinks [2]. Conventional synthetic polymers have the advantages of excellent electrical insulation, good processability, and being lightweight, making them suitable for substrates of thermal interface materials. Among the available polymeric matrixes for TIMs, thermoplastic polyurethanes (TPU) are attractive due to their highly versatile and unique properties. TPU is a kind of multiphase block copolymers, the thermomechanical properties can be easily tailored by changing the molecular chain structure of the soft and hard segments, and the recyclability of thermoplastics gives it an added advantage [3,4]. Unfortunately, the thermal conductivity of most conventional polymeric substrates is very low, in the range of 0.1–0.5 W m^−1^K^−1^ [5]. Ren et al. claimed that the thermal conductivity of laying graphite films/carbon fiber fabrics/TPU composite is up to 242 W m^−1^ K^−1^ at room temperature [6]. In addition, Dong et al. reported that the thermal conductivity and thermal stability were also found to be enhanced by introducing carbon black into TPU matrix [7]. However, most reports have only academic significance, and difficultly can be applied to electronic devices due to their extremely high electrical conductivity, leading device to malfunction because of electron leakage. Hence, the introduction of a thermally conductive but electrically non-conductive inorganic ceramic filler into the polymeric matrix would be one of the promising solutions [8,9,10]. Many studies have reported various methods to develop high thermally conductive and electrically insulating polymer-based composites which include the addition of various ceramic fillers such as boron nitride (BN), aluminum oxide (Al_2_O_3_), aluminum nitride (AlN), silicon carbide (SiC), silicon dioxide (SiO_2_), and zinc oxide (ZnO) into the polymeric matrix [11,12,13,14,15,16,17,18]. Among these ceramic fillers, hexagonal boron nitride (*h*-BN) with a two-dimensional (2D) layered structure stands out owing to its excellent thermal conductivity (250–300 W m^−1^ K^−1^), low thermal expansion coefficient, stable crystal structure, low dielectric constant, high resistivity, and non-toxic properties [19,20]. Liu et al. reported that high thermal conductive *h*-BN filled TPU composites can be enhanced with 2-time by controlling the alignment level of *h*-BN through a fused deposition modeling 3D printing technique, but it is only limited along the printing direction [21].

Interfacial thermal resistance between the polymer matrix and filler is a key factor that influences the thermal conductivity of the composites as known from the literature [22]. The thermal energy in the system is mainly transmitted through the lattice vibrations (phonons) therefore, the discontinuous coupling between the polymer and filler causes phonon scattering, resulting in thermal resistance [5]. Several common solutions have been used to resolve the problem of interfacial thermal resistance. For example, surface functionalization or modification of the filler can create a bonding to improve the adhesion between the filler and polymer matrix that can reduce the intensity of phonon scattering [23,24]. However, some surface functionalization methods are challenging to perform and the effect of these methods in improving the thermal conductivity of composite materials is still limited [25,26,27]. Of late, many studies have reported various preparation methods for establishing a three-dimensional heat conduction network in the composites to deal with the issue mentioned above [27,28,29,30]. Through the compact network structure of continuous thermally conductive fillers, the effect of phonon scattering can be reduced which in turn reduces the interfacial thermal resistance thereby achieving higher thermal conductivity. On the other hand, it also provides continuous heat conduction paths in multiple dimensions and allows heat energy transfer throughout the network [31]. Indeed, these methods have their merits, however, some are time-consuming and complicated, limiting their use in large-scale industrial manufacturing and practical applications [32]. In general, increasing the filler content favors the establishment of a continuous thermal network structure in the composite materials. Nonetheless, adding in excess leads to problems such as the reduced processability of the composite and increased cost of the filler [33,34]. The introduction of a hybrid filler is one other promising strategy for enhancing the composites since it combines the advantages of different filler systems, such as their aspect ratios, geometric dimensions, etc. Fillers along with the polymer matrix forms a complex three-dimensional (3D) thermal network structure, consequently improving the performance and lowering the manufacturing cost of the composite materials [35,36,37,38,39].

During the preparation of thermally conductive polymer composites, the dispersion of fillers is one of the key factors that affects thermal conductivity [40]. Simple mixing strategies inevitably lead to an uncontrolled distribution of fillers, which might limit the synergistic enhancement between the fillers in the heat transfer network construction process [32]. Several methods have been reported in the literature that assist in resolving the uncontrolled distribution problem. Several methods have been reported in the literature that assist in resolving the uncontrolled distribution problem, such as solution compounding, roll mixing, and melt-compounding [41,42,43]. Among them, the melt-compounding is the method commonly utilized in the batch manufacturing of thermoplastic composites since it is a continuous process and involves simple operating methods wherein the fillers can be uniformly distributed in the continuously produced polymer matrix. However, when undergoing the screw extrusion process, the viscosity of composite influences the distribution as the high shear force generated during the mixing might damage and crack the thermally conductive fillers making it more challenging to manufacture on a large scale for industrial applications [44].

In this work, we propose a facile and effective method to prepare thermally conductive composite constituting compact and continuous fillers. Firstly, the spheroidized three-dimensional functional hybrid fillers, Al_2_O_3_/*h*-BN (ABN), were prepared by mechanical mixing and spray drying processes [45,46]. Secondly, the ABN functional hybrid fillers were uniformly mixed with the TPU matrix through the melt-compounding process to form the ABN/TPU thermally conductive composite which was then made into a pellet by hot pressing. The results indicate that the thermal conductivity of ABN/TPU composites is substantially improved from the synergistic association of Al_2_O_3_ nanoparticles filled with *h*-BN. At 30 wt.% of filler content, the ABN/TPU thermally conductive composites can reach a high thermal conductivity of 1.39 Wm^−1^ K^−1^ and can considerably reduce the amounts of *h*-BN. It is confirmed that ABN functional hybrid thermal fillers can form a continuous three-dimensional (3D) thermal network structure in the TPU matrix and maintain the network framework during composite preparation. The method claimed in this study is facile, cost-effective and therefore offers new possibilities for the large-scale production of thermally conductive composite materials constituting a 3D thermal network of hybrid fillers with commercial applications in thermal interface materials.

## 2. Materials and Methods: Experimental

### 2.1. Materials

The *h*-BN powder with a particle size of 2–3 μm was provided by National Nitride Technologies Co., Ltd. (Taichung, Taiwan). Al_2_O_3_ nanoparticles with a particle size of 30–50 nm were purchased from Yong-Zhen Technomaterial Co., Ltd. (Taipei, Taiwan). Thermoplastic polyurethane (TPU, Elastollan S 85A) was provided by BASF Co., Ltd. (Ludwigshafen, Germany). All chemicals used in the experiment are of analytical grade and without any further purification.

### 2.2. Preparation of Al_2_O_3_/h-BN Spherical Three-Dimensional Functional Hybrid Filler

**Figure 1:**
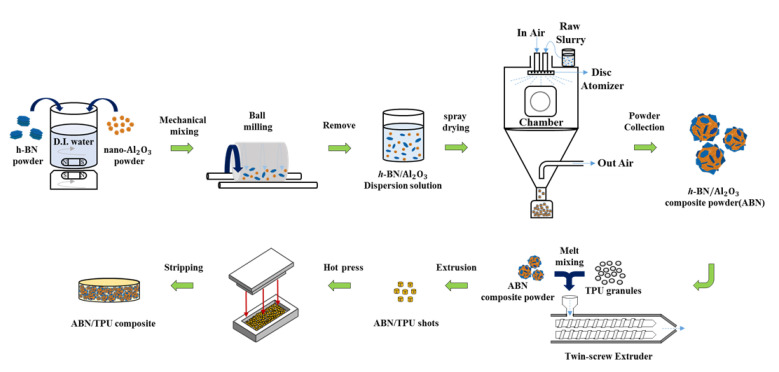
Schematic diagram of preparation of spherical *h*-BN/Al_2_O_3_ functional hybrid thermal conductive filler (ABN) and thermoplastic polyurethanes (TPU) thermally conductive composites.

Mechanical mixing and spray drying methods were used for the preparation of the ABN hybrid filler. At first, the Al_2_O_3_ nanoparticles were uniformly mixed with *h*-BN powder to form the starting slurry via mechanical mixing. The Al_2_O_3_ nanoparticles and *h*-BN powders in the ratio of 1:1 (6.7 kg) were dispersed in de-ionized water (5.4 L). After continuous agitation with the magnetic stirrer, the components were thoroughly mixed in the vertical ball mill to attain a uniform Al_2_O_3_/*h*-BN solution. The above-formed dispersion was then transferred to the spray drying system (CNK SDDNH-3, GUS Technology Co., Ltd., Taipei, Taiwan), from which the Al_2_O_3_/*h*-BN spherical three-dimensional functional hybrid thermal conductive filler (ABN) was obtained. The processing parameters of spray drying are described as below: The temperature ranges for in air and out of air were set at 200 to 220 °C and 60 to 80 °C respectively. The temperature of the chamber was set at a range of 90 to 130 °C. The press air was set at 1.5 kg/cm^2^, the disc rotation speed was set at 20,000 rpm, and the feed rate was fixed at 3 kg/h. The experimental procedure is shown in Figure 1.

### 2.3. Composite Preparation

ABN/TPU thermally conductive composites were prepared by melt-compounding. Firstly, the ABN functional hybrid filler with different loading percentages was blended with the TPU matrix at a melting temperature of 180 °C for 10 min using a twin-screw extruder (Kobelco Co., Ltd., Tokyo, Japan). After extrusion, the composite samples were cut into shots and dried in the hot-air oven at 100 °C for 24 h after which the composite pellets with a thickness of 3 mm were hot-pressed at 180 °C with 4 MPa pressure by a Vacuum Type Heating Pressure Shaping Machine (Long Chang Co., Ltd., Tainan, Taiwan). To validate that the compact and continuous structure of ABN fillers could assist in improving the thermal properties of the composites, the same method was applied to prepare *h*-BN/TPU with different filler loading percentages as the control sets. In this work, the weight percentage of filler loading in ABN/TPU and *h*-BN/TPU composites were designated at 10 wt.%, 20 wt.%, and 30 wt.%.

### 2.4. Characterizations of Composite Materials

The crystal structure of fillers and composite materials were characterized by X-ray diffraction (EMPYREAN, Panalytical Co., Ltd., Almelo, The Netherland) using Cu Kα radiation (λ = 1.54 Å) as a X-ray source in the 2*θ* range of 10°–80° with a step size and scan speed of 0.05° and 2°/min respectively. Fourier transform infrared spectrometer (JASCO FT/IR-4150, Tokyo, Japan) was used to analyze the chemical structure of thermally conductive TPU composites and fillers. The vibration spectra were acquired from 400 to 4000 cm^−1^. The top and cross-section view of microstructures of the prepared ABN functional hybrid filler and thermally conductive composites were observed through Field Emission Scanning Electron Microscopy (FE-SEM, JSM-7610F, JEOL, Tokyo, Japan). Energy dispersive spectroscopy (EDS) was performed using the FE-SEM equipped with an EDS detector to assess the ABN functional hybrid fillers. Thermogravimetric Analyzer (STA7300, Tokyo, Japan) was used to estimate the filler content of ABN in the ABN/TPU composites. Thermal degradation evaluation of all the samples was performed from 25 °C to 900 °C with a heating rate of 10 °C/min under the nitrogen atmosphere. Based on the transient plane source method (TPS), the thermal constants analyzer (Hot Disk TPS 2500, Gothenburg, Sweden) was used for understanding the thermal conductivity of the composite and all measuring processes were based on the ISO 22007-2 standards [29,32,33,47]. Infrared thermography (IR-TCM HD, Jenoptik AG, Jena, Germany) was used to record the change in the surface temperature of the TPU composite during heating time. The tensile properties were measured on a universal tensile machine (Instron4464, Instron Corporation, Norwood, MA, USA) at a cross-head speed of 5 mm/min according to ASTM D412. All the measurements were carried out at room temperature (25 °C). For each composition, the average value of 5 specimens was reported. At least 5 samples were tested for each composite.

## 3. Results and Discussion

**Figure 2:**
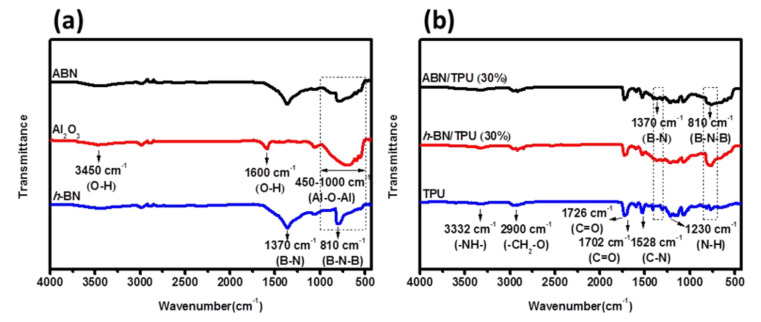
FTIR spectrum of (**a**) *h*BN powders, Al_2_O_3_ NPs, and aluminum oxide/hexagonal boron nitride (ABN) functional hybrid fillers and (**b**) pure TPU, *h*-BN/TPU and ABN/TPU composites with 30 wt.% filler loading, respectively.

The ABN functional hybrid filler was dispersed in the TPU matrix to form the thermally conductive composite. The FTIR was utilized in understanding the formation of the composite (30 wt.% of filler loading) with the preliminary identification of their chemical composition. Figure 2a shows the FTIR spectrum of *h*-BN powders, Al_2_O_3_ nanoparticles, and Al_2_O_3_/*h*-BN (ABN). The pure *h*-BN exhibits two sharp characteristic peaks at wavenumbers 1370 cm^−1^ and 810 cm^−1^, indicating B-N in-plane stretching mode and B-N-B out-of-plane bending mode, respectively [20]. From the spectrum of Al_2_O_3_ nanoparticles, the vast broadband at the wavenumber ranging from 400 cm^−1^ to 1000 cm^−1^ can be attributed to the Al-O-Al stretching vibration. In addition, two characteristic peaks are observed at wavenumbers 1600 cm^−1^ and 3400 cm^−1^. Those two peaks respectively correspond to –OH groups bending mode and hydroxyl group (–OH) stretching mode of the absorbed water [48]. The spectrum of ABN is mainly dominated by the characteristic *h*-BN peak comparable to that of the *h*-BN spectrum. Correspondingly, the characteristic absorption broadband of alumina is seen at wavenumbers ranging from 400 cm^−1^ to 1000 cm^−1^, verifying that the Al_2_O_3_ nanoparticles and *h*-BN powders were successfully blended to produce ABN through the spray drying process.

Figure 2b displays the FTIR spectrum of pure TPU, *h*-BN/TPU (30 wt% filler loading), and ABN/TPU (30 wt.% filler loading) composites. In the pure TPU spectrum, the broadband observed at 3332 cm^−1^ corresponds to amine (-NH-) groups. Another broadband located at around 2900 cm^−1^ which is split into multiple peaks can be attributed to the –CH_2_-O stretching mode. Two sharp peaks at 1726 and 1702 cm^−1^ are correlated to the ester carbonyl group and the carbonyl stretching of the urethane groups. In addition, peaks at 1530 cm^−1^ and 1230 cm^−1^ correspond to urethane C-N stretching and N-H bending absorption, respectively [49]. The characteristic peaks of *h*-BN can be found in both the *h*-BN/TPU and ABN/TPU spectra, however, the intensity gets weaker due to the overlapping of the TPU and high-intensity additives peaks [27]. The overall FTIR results suggest that the inorganic fillers are not chemically linked but are physically attached to the TPU polymer and therefore there is no disappearance or appearance of new bonds in the *h*-BN/TPU and ABN/TPU composites [50].

**Figure 3:**
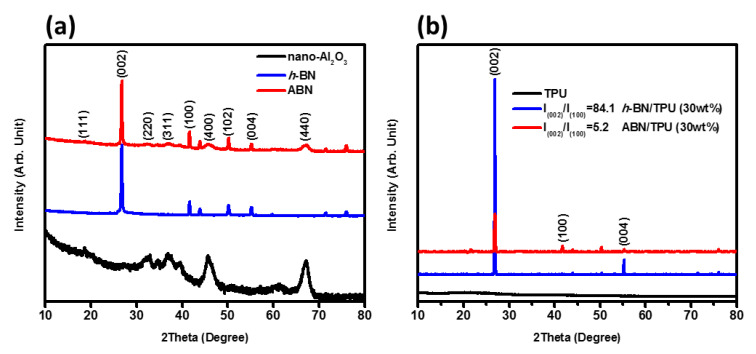
XRD patterns of (**a**) *h*-BN, Al_2_O_3_, and ABN functional hybrid fillers. (**b**) *h*-BN/TPU and ABN/TPU composites with 30 wt.% filler loading, respectively.

The evolution of the structure of each sample can be realized from the XRD patterns shown in Figure 3a. The main peaks of the pure *h*-BN can be observed at 2*θ* = 27.11°, 41.95°, 50.50°, and 55.35° corresponding to the (002), (100), (102), and (004) planes, respectively that belong to the characteristic hexagonal crystal structure (JCPDS:85-1068) [34,51]. The crystallinity of Al_2_O_3_ nanoparticles can also be reflected in the XRD pattern. The main peaks located at 2*θ* = 19.43°, 31.99°, 37.70°, 45.88°, and 66.89°, can be assigned to the (111), (220), (311), (400), and (440) planes, respectively that matches well with the hexagonal phase of Al_2_O_3_ belonging to the *R-3c* space group (JCPDS:00-010-0425) [52]. It is noticeable that the patterns correspond to the typical pure *h*-BN and Al_2_O_3_ structures, and no other impurity phases or heterostructures can be detected according to the MDI Jade database. This demonstrates the *h*-BN and Al_2_O_3_ samples prepared are of high purity.

The XRD pattern of the ABN functional hybrid filler is composed of the characteristic peaks of *h*-BN and Al_2_O_3_, as seen in Figure 3a with no other additional peaks inferring that the ABN functional filler can be effectively prepared by the spray drying process and is in accord with FTIR studies, as discussed in Figure 2a. The XRD patterns of the TPU, *h*-BN/TPU, and thermally conductive ABN/TPU are shown in Figure 3b. The broad diffraction peaks at around 2*θ* of 15° and 30° designate the amorphous property of the TPU polymer. The presence of all the characteristic peaks of *h*-BN and ABN in the *h*-BN/TPU and ABN/TPU diffraction patterns denote, and with no obvious position shift, which suggested that the crystal structures of thermally conductive composites are not affected by the processing method. It is interesting to note that high-intensity *h*-BN peak dominates in both patterns. As verified by several research works, the degree of the orientation of *h*-BN sheets in the polymer matrix is one of the essential factors affecting the thermal conductivity and this feature can be examined by XRD analysis. The (002) and (004) planes are attributed to the horizontally oriented BN, while the (100) plane is due to vertically oriented *h*-BN [53,54]. As seen in Figure 3b, the two strong peaks at 2*θ* = 26.5° and 54.7° of *h*-BN/TPU composites representing (002) and (004) resulted from the horizontal orientation of the *h*-BN sheets that was induced by hot-pressing with a perpendicular pressure. On the other hand, in the XRD pattern of ABN/TPU composite, the (100) plane appeared while the intensity of (002) and (004) substantially reduced. This phenomenon indicates that the orientation of the *h*-BN sheets inside the ABN/TPU composites is random, contributing to a higher intensity of (100) plane, which are in accord with SEM analysis as discussed in the next section [29,55].

In addition, the intensity ratio of (002) and (100) characteristic peaks could be used to evaluate the orientation degree of *h*-BN filled in the polymer matrix. As verified by several research works [20,53], the more vertically arranged *h*-BN structures can be created when the value of I_(002)_/I_(100)_ is lower. The I_(002)_/I_(100)_ ratio of ABN/TPU composites and *h*-BN/TPU composites are 5.2 and 84.1, respectively. The I_(002)_/I_(100)_ value of *h*-BN/TPU composite is 16 times higher than the ABN/TPU composite, indicating that a random structure of a *h*-BN-filled composite by a hot press process is complicated since *h*-BN sheets tend to distribute in the horizontal direction. Creating a random structure of a *h*-BN-filled composite by a hot press process is complicated since *h*-BN sheets tend to distribute in the horizontal direction. However, through the process presented in this work, more vertically arranged *h*-BN structures can be created, which is beneficial to the ABN filler in the composite material in order to form the effective continuous heat conduction chains to provide more heat conduction paths.

**Figure 4:**
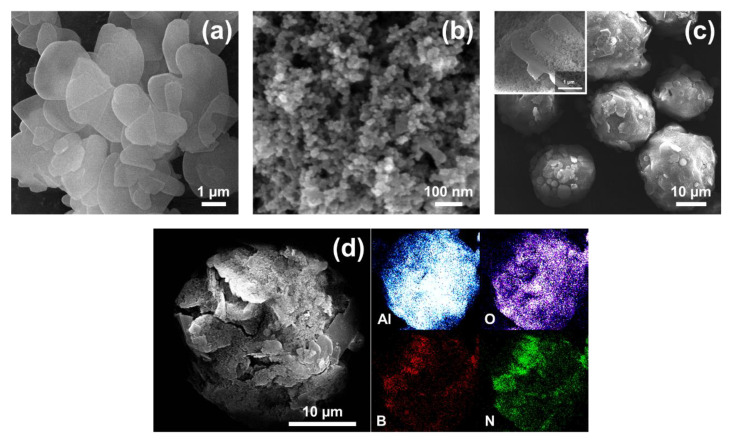
FE-SEM images of (**a**) *h*-BN, (**b**) Al_2_O_3_, and (**c**) ABN functional hybrid fillers and the inset shows the morphology of ABN particles surface at a higher magnification, and (**d**) cracked surface and EDS elemental mapping of the ABN functional hybrid filler.

The surface morphology of the pure *h*-BN powder, Al_2_O_3_ nanoparticles, and ABN functional hybrid filler was studied by field emission scanning electron microscopy (FE-SEM). In Figure 4a, it can be seen that the *h*-BNs displayed a hexagonal and plate-like shape with an average particle size of 3 *μ*m. A uniform spherical shape of Al_2_O_3_ NPs with the particle size of the nanospheres ranging from 30 to 50 nm can be seen in Figure 4b. The surface morphology of the ABN functional hybrid filler after spray drying, displayed in Figure 4c, shows that most of the ABN particles form the spherical-like structures with their particle sizes ranging from 10 and 40 *μ*m. At a higher magnification, as demonstrated in the inset of Figure 4c, the ABN particles have a rough surface, which is due to the decoration of Al_2_O_3_ NPs on the surface. In conventional theory, if the components powders are approximately of the same size, they would tend to uniformly distribute in the composite particles. However, when the components powders are composed of two different particle sizes, the radial segregation of particles occurs. Through the Brownian motion, smaller particles with the higher mobility occlude larger particles and therefore, according to this mechanism, surrounding or a coating of one component by others can be created [46,56,57].

To further reveal that the *h*-BN powder surface was wrapped by compact Al_2_O_3_ NPs layers, ABN particles were compressed to form a crack on the surface, and the results were confirmed by the elemental mapping images as shown in Figure 4d. As expected, the abundant Al and O elements exhibited uniform and continuous distribution throughout the ABN particles demonstrating the Al_2_O_3_ NPs coating on the *h*-BN powders. Besides, a considerable amount of Al_2_O_3_ NPs is located between the neighboring *h*-BN particles that serve as bridges. This kind of unique structure is conducive to form a more efficient 3-D thermally conductive networks.

**Figure 5:**
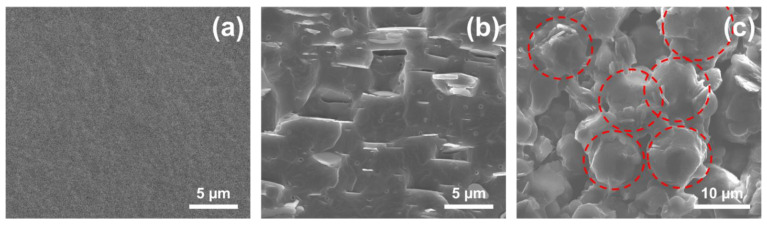
Cross-sectional images of thermally conductive composites and pure TPU: (**a**) Pure TPU, (**b**) *h*-BN/TPU, and (**c**) ABN/TPU composites with 20 wt.% filler loading, respectively.

Figure 5a–c presents the cross-section images of the samples. All the samples underwent brittle fractures after being immersed in liquid nitrogen. The dispersion and structure distribution between fillers and polymer can be observed in these figures. Figure 5a shows the cross-section image of pure TPU. Because of the brittle fracture, the surface of the pure TPU is clean and smooth. In contrast, as seen in Figure 5b,c, incorporated hybrid functional filler composites exhibit a rough surface and crumpled fracture structure with many embedded particles, a result of the local polymer deformation that occurred due to cracking from the addition of the hybrid functional fillers [58]. Despite the rough surface, thermally conductive fillers show a uniform dispersion and homogeneity with no large clusters in the matrix. This is attributed to the state of particle dispersion according to the melt mixing method, as mentioned in the introduction. Most of the thermally conductive filler particles play an essential role in constructing the thermal conductive pathways, and they have higher thermal conductivity along the direction of the heat flow [53]. We further compared the cross-sectional morphologies of TPU composites filled with the different weight percentage of thermally conductive/functional fillers. Figure 5b,c displays the images of *h*-BN/TPU and ABN/TPU composites with the filler loading of 20 wt.%. As seen in Figure 5b, the *h*-BN sheets in *h*-BN/TPU composite film are almost horizontally oriented as rendered by hot-pressing with perpendicular pressure, signifying that the thermal conductivity could be quite different in various directions, thereby limiting its use in practical applications since heat dissipation between the devices and heat sinks usually occurs in the vertical direction [55]. In addition, from the figure, we observed that at a low weight percentage (20 wt.%) of *h*-BN loading, fillers are unable to create a continuous heat flow path with lower interfacial thermal resistance. Due to this inherent problem, a large amount of thermally conductive fillers is required to establish a thermally conductive pathway network structure, and hence increases the cost of fillers.

On the other hand, as shown in Figure 5c, the spherical ABN hybrid functional hybrid fillers, proposed in this work, are connected to form a continuous thermally conductive pathway network (marked in red), which plays a pivotal role in enhancing the thermal conductive of the composite. The most significant advantage of spherical ABN functional hybrid filler is that it has no specific orientation after being processed by hot pressing. The spherical structure provides continuous pathways in all dimensions and ensures most of the energy is being transferred through the filler networks [59]. Therefore, compared with the *h*-BN filler from our previous work, the spherical ABN functional hybrid filler possesses a more continuous structure in all dimensions and longer range, resulting in a significant improvement of composite thermal conductivity despite the low filler concentration. As the above mentioned, self-assembled 3-D network in TPU is successfully generated by the insertion of the Al_2_O_3_/*h*-BN hybrid through our designed method. Moreover, our designed method is not only facile, but also offers new chances for large-scale production.

**Figure 6:**
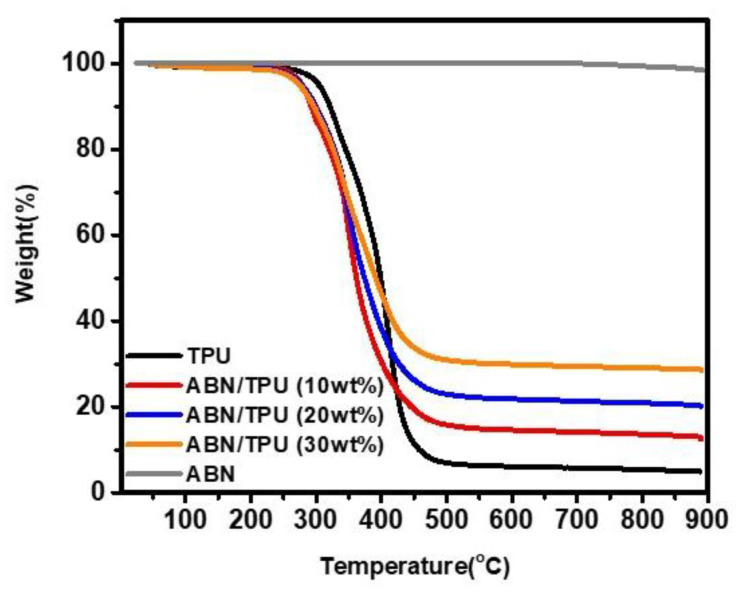
TGA curves of the ABN filler, ABN/TPU composites, and pure TPU.

Thermal stability is crucial for polymer materials, which is the limiting factor in both processing and applications. In this work, the content of ABN in the composites and the thermal stability of the composites were verified by Thermogravimetric Analyzer (TGA) tested at a heating rate of 10 °C min^−1^ from 25 °C to 900 °C under the nitrogen atmosphere. Figure 6 demonstrates the TGA curves of the ABN filler, the pure polymer TPU, and ABN/TPU composites loaded with different ABN contents. The results show that the ABN functional hybrid fillers prepared by our method exhibited high thermal stability with no significant weight loss up to 900 °C. Compared to ABN filler, pristine TPU, and ABN/TPU composites demonstrate two main degradation stages as seen in the weight loss curve. The first stage is between 300 °C–350 °C, which is attributed to the cleavage of the urethane linkage to polyol and isocyanate in the TPU hard segment [60]. The second stage is between 350–480 °C, which is ascribed to the cleavage of the polyol and diisocyanate into smaller molecules in the TPU soft segment [61].

The residual weight of ABN/TPU composites is higher than that of pure TPU which completely degrades at around 900 °C. The content of ABN filler can be obtained from the residual weight at 900 °C and hence from calculating the differences in the residual weight, the addition of about 10 wt.%, 20 wt.%, and 30 wt.% ABN to the TPU substrate can be confirmed.

**Figure 7:**
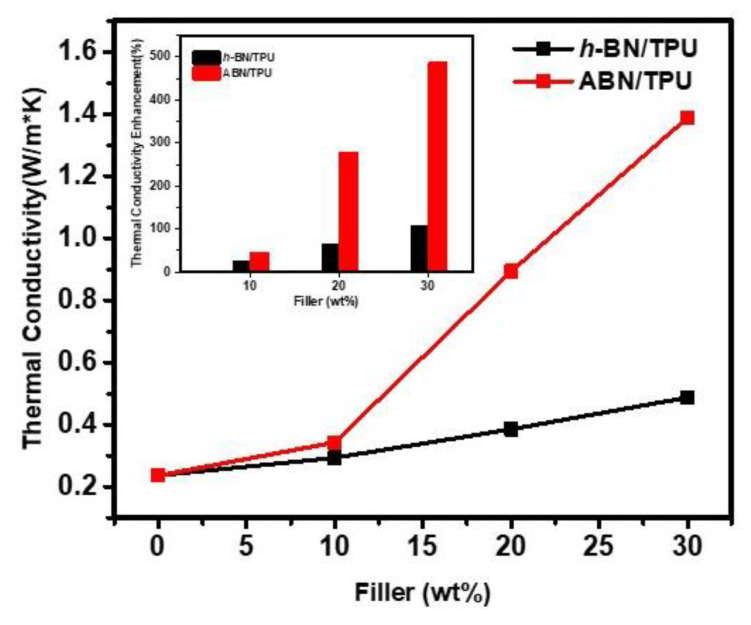
Thermal conductivity of *h*-BN/TPU and ABN/TPU composites versus filler loading (wt.%). The inset shows the comparison of the thermal enhancement factor among different composite samples.

Figure 7 shows the thermal conductivity of *h*-BN/TPU and ABN/TPU composites with different filler loadings ranging from 0 wt.% to 30 wt.%, respectively. Owing to its amorphous structure and phonon scattering, the thermal conductivity of the pristine TPU is extremely low, which is at around 0.2 W m^−1^ K^−1^. For all composites, the thermal conductivity remarkably improved with the increasing content of the fillers. However, the enhancement in the thermal conductivity of the composites with different fillers showed a distinct difference. As clearly seen in Figure 7, the ABN/TPU composite showed higher thermal conductivity than the *h*-BN/TPU with the same filler loading. For example, with 30 wt.% filler loading, the thermal conductivity of ABN/TPU composite is 1.39 W m^−1^ K^−1^, while the *h*-BN/TPU composite showed a trifling thermal conductivity of 0.48 W m^−1^ K^−1^ which is three times lower, signifying that the spherical ABN functional filler obtained by spray-drying demonstrated a greater advantage in improving the thermal conductive properties of the composite.

The trend observed in the thermal conductivity of ABN/TPU composites is nonlinear. With the 10 wt.% filler loading, the thermal conductivity is 0.34 W m^−1^ K^−1^ showing an improvement of around 45%. Beyond the threshold for percolation (between 10 wt.% and 20 wt.% of the filler loading), this value sharply increases [27,29]. When the content of spherical ABN particles is further increased to 30 wt.%, the thermal conductivity achieved is 1.39 W m^−1^ K^−1^, equivalent to a dramatic upsurge of 488% compared to that of the pure matrix. The variation in the thermal conductivity acquired from the ABN/TPU composites can be explained by the network structure of the fillers and distribution state in the TPU matrix. The polymer matrix is interposed between the adjacent fillers under low filler loading, disrupting the contact between the particles. This results in the phonon scattering, further increasing the interface thermal resistance, which finally leads to reduced heat conduction. When the filler content is continually increased beyond the threshold for percolation (between 10 wt.% and 20 wt.%), the increasing number of spherical ABN fillers contact with the adjacent ones, forming a densely packed structure that facilitates phonon transfer in a continuous thermal network.

**Figure 8:**
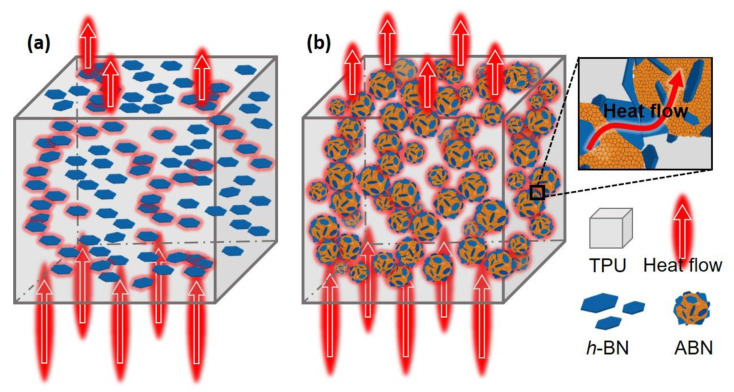
Schematic of two types of the composites’ heat flowing paths: (**a**) *h*-BN/TPU and (**b**) ABN/TPU, respectively.

To better illustrate the effect of different fillers on the formation of thermally conductive pathways, two models with the efficient network for heat flow in the composites were proposed as shown in Figure 8. Usually, to obtain high thermal conductivity, a heat flow channel along the heat flow direction should be generated. However, the *h*-BN/TPU composites seen in Figure 8a comprises of a few thermally conductive pathways, and many *h*-BN sheets are not involved in the construction of pathways. The horizontal orientation of *h*-BN sheets can be formed after vertical hot pressing, which results in the interruption of heat transfer along the vertical direction, but nonetheless, the thermal conductivity cannot be effectively improved. In contrast, the spherical ABN particles are linked to each other to form a continuous three-dimensional network structure for unhindered heat flow in the TPU polymer as shown in the schematic diagram in Figure 8b. These spherical ABN particles can not only retain the structure after hot pressing but are also beneficial in achieving the dense packing of the particles to form thermally conductive paths with reduced interfacial thermal resistance, thereby enhancing the phonon transfer. The heat transfer always alternates between fillers and polymers when fillers randomly distributed in the composites, hence, the interface thermal resistance has a very bad effect on the improvement of thermal conductivity. The synergistic enhancement of the spherical ABN fillers on thermal conduction can also be clearly observed, in which the Al_2_O_3_ NPs are connected to the neighboring *h*-BN platelets like bridges to construct the continuous phonon transmission pathways. Therefore, this demonstrates that the spherical ABN functional filler prepared in this work is promising in achieving a high thermal conductivity in polymer composites.

**Figure 9:**
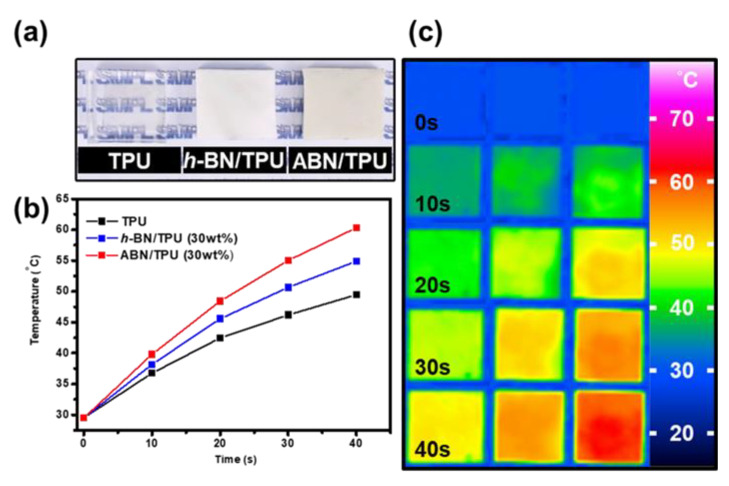
(**a**) Photographs of the pure TPU, *h*-BN/TPU, and ABN/TPU composite. (**b**)The surface temperature variation with time of pure TPU, *h*-BN/TPU, and ABN/TPU composites during heating. (**c**) Infrared thermal images of pure TPU, *h*-BN/TPU, and ABN/TPU composites (from left to right) with 30 wt.% filler concentration.

The heat transfer capability of *h*-BN 30 wt.% loading and ABN 30 wt.% loading in the TPU composite materials were tested by heating on an electric hot plate for 40 s and analyzing the temperature response, recorded by the infrared thermography as shown in Figure 9. Figure 9a presents the photographs of the pure TPU, *h*-BN/TPU, and ABN/TPU composites. The pure TPU exhibits high transparency, while the *h*-BN/TPU and ABN/TPU composites are opaque and white. The heating curves of the samples are shown in Figure 9b, and the images of infrared thermography at different stages of the three samples mentioned above are illustrated in Figure 9c. The surface temperature changes with an increase in the heating time and the color of all the samples gradually change from blue to red. It can be seen that the rate of change in the surface temperature of all composites is faster than that of pure TPU. Figure 9b,c shows the heat transfer trend of the three samples when heated from 25 °C to 65 °C on the hot plate. After heating for 40 s, it is apparent that the increase in the surface temperature of the ABN/TPU was the fastest, and the surface temperature of ABN/TPU was the highest. The addition of spherical ABN fillers for enhancing the thermal conductivity of composites is consistent with the order of their thermal conductivity values as mentioned above. From the IR results, it can be confirmed that through ABN/TPU composites prepared by the proposed method, the continuous thermal network can be formed and heat transfer occurs more effectively.

**Figure 10:**
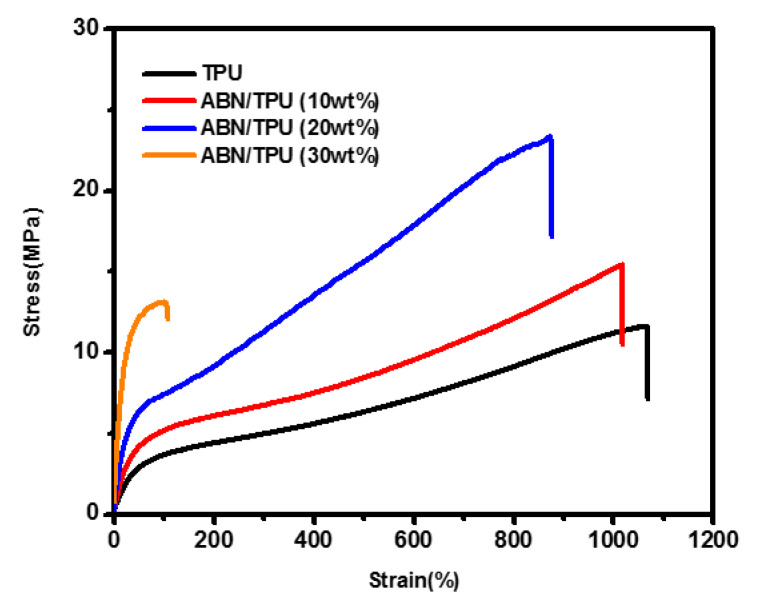
Stress-strain dependence of pure TPU and TPU composites with different contents of ABN filler.

To further realize the structure and interfacial properties between the ABN functional filler and TPU, the mechanical properties of ABN/TPU composites were investigated by using a tensile stress-strain test. The tensile stress-strain curves of pure TPU, and TPU composites with various ABN filler loading ranging from 10 wt.% to 30 wt.% are shown in Figure 10. A typical tensile behavior of an elastomer can be observed in the pure TPU. The tensile strength and strain-at-failure of the pure TPU was 82 MPa and 570%. While adding the ABN functional filler, the tensile strength of TPU composites were firstly promoted, then decreased with the increase in the filler loading. The maximum tensile strength of 21 MPa can be reached when the filler loading is 20%, which is about three times that of the pure TPU matrix, and the elongation was only slightly decreased. This strengthened tensile stress is due to the intrinsic favorable mechanical property of ABN functional fillers and strongly interfacial interaction with the TPU matrix [62,63]. When subjected to stress, the TPU chains were initially stretched along the stress direction, then the external stresses applied to the composite can be shared efficiently by transferring to the filler via the interface interaction between the filler and TPU chain [51]. Furthermore, the filler has a higher tensile strength than those of the TPU matrix and can act as a skeleton to help the matrix to bear the load, resulting in the higher tensile stress of composites. However, the further increase of ABN functional filler content to 30 wt.%, results in both sharply decreased tensile strength and elongation at the break of the composites, and exhibited in obvious brittle fracture characteristics, a phenomena that can be attributed to the aggregation of the filler, which weakened the encapsulating and supporting role played by the TPU matrix, indicating that the presence of the ABN functional filler is unfavorable for maintaining the tensile ductility of the composite, especially at an extremely high content [29,60]. The above results demonstrated that the improvement of thermal conductivity in the TPU composites is due to the strong coupling in the interface of the ABN filler and TPU, which are beneficial in achieving and forming a densely continuous three-dimensional network for thermal conduction.

## 4. Conclusions

In this work, a novel spherical hybrid filler (ABN) containing Al_2_O_3_ NPs and *h*-BN was designed through simple mechanical mixing and spray drying processes. This filler was utilized to prepare the TPU composite matrix with the continuous three-dimensional (3D) thermal conduction network. The ABN/TPU composites prepared by melt mixing and hot compression was compared with *h*-BN/TPU composites in terms of their thermal conductivity wherein the ABN/TPU composite exhibited thermal conductivity of 1.39 Wm^−1^ K^−1^ with a filler loading of 30 wt.%, which is six times higher than that of pure TPU and three folds elevated than that of the *h*-BN/TPU composite. Enhancement in the thermal conductivity could be attributed to the three-dimensional (3D) thermal conduction network. As the filler, ABN particles provided a continuous heat conduction path while reducing the interface thermal resistance of the matrix. SEM images confirmed that Al_2_O_3_ NPs were located between neighboring *h*-BN powders and served as bridges, which in turn assisted to build a continuous phonon transmission path. The above results can provide new insights into the construction of filler-contained composites with 3D isolation networks and demonstrated strong potential for the design of large-scale manufacturing of thermal interface materials.

## Data Availability

Data sharing not applicable.
